# Site-specific *N*-glycosylation analysis of soluble Fcγ receptor IIIb in human serum

**DOI:** 10.1038/s41598-018-21145-y

**Published:** 2018-02-09

**Authors:** Hirokazu Yagi, Daisuke Takakura, Lubka T. Roumenina, Wolf Herman Fridman, Catherine Sautès-Fridman, Nana Kawasaki, Koichi Kato

**Affiliations:** 1 Faculty and Graduate School of Pharmaceutical Sciences, Nagoya City University, 3-1 Tanabe-dori, Mizuho-ku, Nagoya, 467–8603 Japan; 2 Department of Medical Life Science, Graduate School of Medical Life Science, Yokohama City University, Suehiro-cho 1-7-29, Tsurumi-ku, Yokohama, 230–0045 Japan; 3 UMRS1138, Université Paris Descartes, Université Pierre et Marie Curie, 15, rue de l’Ecole-de-Médecine, 75270 Paris, France; 4 Institute for Molecular Science and Okazaki Institute for Integrative Bioscience, National Institutes of Natural Sciences, 5-1 Higashiyama Myodaiji, Okazaki, 444–8787 Japan; 5 Present Address: Center for Integrated Medical Research, Keio University School of Medicine, 35 Shinanomachi, Shinjuku-ku, Tokyo, 160–8582 Japan

## Abstract

Fc-receptors for immunoglobulin G (FcγRs) mediate a variety of effector and regulatory mechanisms in the immune system. *N*-glycosylation of FcγRs critically affects their functions which is well exemplified by antibody-dependent cell-mediated cytotoxicity (ADCC) and phagocytosis mediated by homologous FcγRIIIa and FcγRIIIb, respectively. Although several reports describe *N*-glycosylation profiles of recombinant FcγRIII glycoproteins, much remains unknown regarding their native glycoforms. Here we performed site-specific *N*-glycosylation profiling of a soluble form of FcγRIIIb purified from human serum based on mass spectrometric analysis. Our data indicate a distinct and common tendency of the glycoforms exhibited at each *N*-glycosylation site between the native and the previously reported recombinant FcγRIII glycoproteins. Among the six *N*-glycosylation sites of serum soluble FcγRIIIb, Asn45 was shown to be exclusively occupied by high-mannose-type oligosaccharides, whereas the remaining sites were solely modified by the complex-type oligosaccharides with sialic acid and fucose residues. The results of our endogenous FcγRIII glycoform analyses are important for the optimization of therapeutic antibody efficacy.

## Introduction

**Figure 1:**
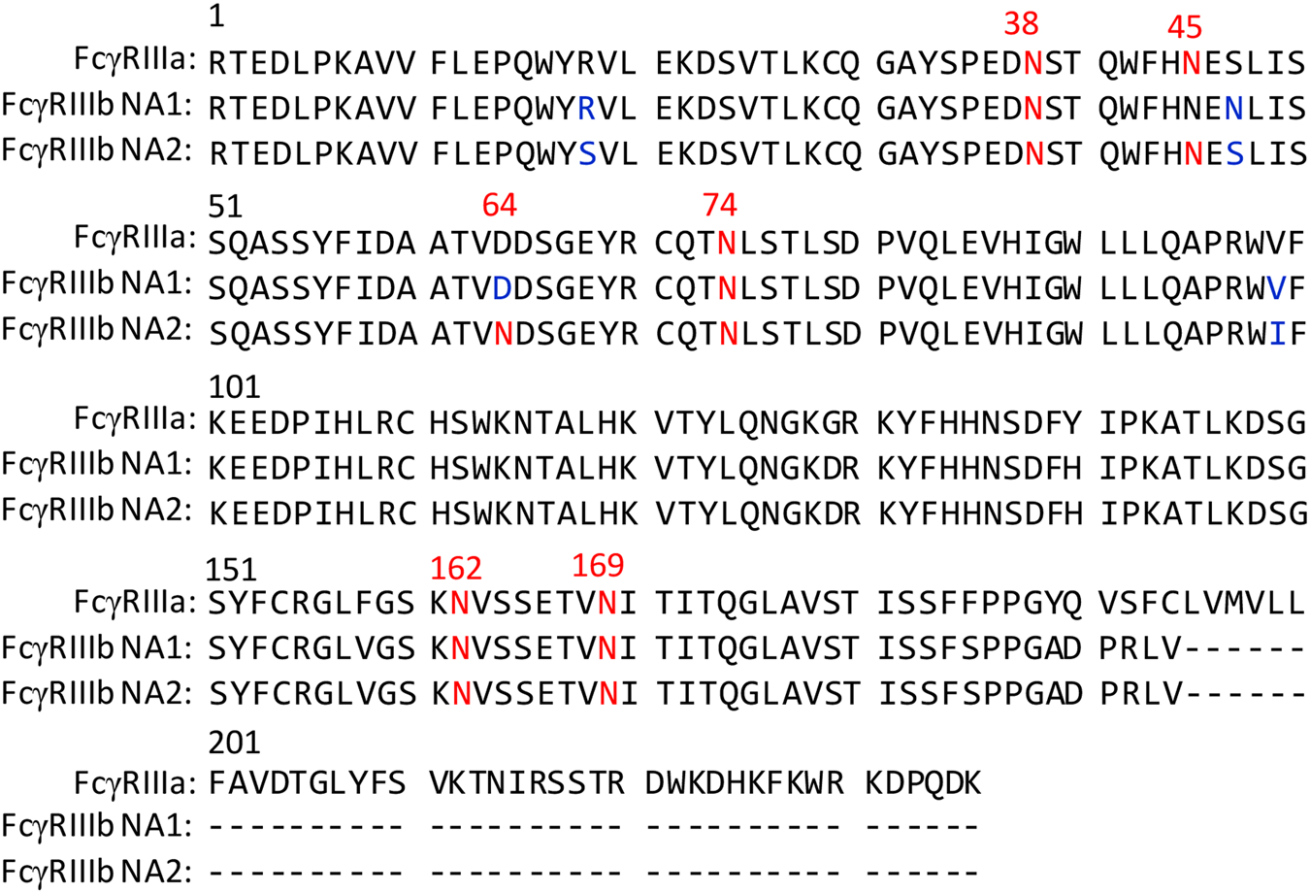
Sequence alignments of the extracellular regions of human FcγRIIIa and sFcγRIIIb NA1 and NA2 forms. *N*-glycosylation sites are shown in red. The residues substituted between NA1 and NA2 are shown in blue.

Various effector and regulatory mechanisms in the immune system are mediated through the interactions between immunoglobulins (Igs) and their cognate receptors that specifically recognize their Fc portions^1–3^. Fc-receptors for IgG (FcγRs) are categorized into three classes: FcγRI, FcγRII, and FcγRIII, which exhibit different binding affinities to IgG isotypes and distinct expression profiles on immune cells. In humans, each FcγR class shows structural variations resulting from multi-genes, alternative splicing, and genetic polymorphisms^4^. Human FcγRIII has two isoforms, transmembrane FcγRIIIa and glycosylphosphatidylinositol-linked FcγRIIIb, encoded by two individual genes, and share 96% amino acid sequence identity in their extracellular Fc-binding regions (Fig. 1). FcγRIIIa is primarily expressed on natural killer cells and promotes antibody-dependent cell-mediated cytotoxicity (ADCC) by interacting with the IgG of the antigen–antibody complex^3,5^, whereas FcγRIIIb is exclusively expressed on neutrophils and mediates the degranulation and phagocytosis of the antibody-labeled target cells^6–8^. These receptors exist not only as membrane proteins but also in soluble forms, designated as sFcγRIIIa and sFcγRIIIb, each comprising two extracellular Ig-fold-domains proteolytically cleaved from the transmembrane segment^9–11^.

FcγRs are modified with N-glycans that significantly affect their interactions with IgGs^12–14^, and human FcγRIIIa molecules with different *N*-glycosylation patterns exhibit different affinities for IgG^15^. This is well exemplified by the effects of glycosylation at Asn45 and Asn162 of human FcγRIIIa on its interactions with IgG1^12,16^. Crystallographic data suggest that the Asn162 glycan has the potential to interact with the *N*-glycan of IgG1-Fc, thereby reinforcing the IgG1–FcγRIIIa interactions, whereas the Asn45 glycan cause steric hindrance to the Fc of IgG1^17–19^. The intermolecular carbohydrate–carbohydrate interactions involving the Asn162 glycan can be optimized to increase the FcγRIIIa-binding affinity of IgG1 by removing the core fucose of the Fc glycan, which offers a promising strategy for the improvement of therapeutic antibody efficacy^20–23^. Hence, the glycosylation of FcγRs is now considered a critical factor in the design and development of antibody therapeutics^13,14,24^.

*N*-glycosylation profiling of FcγRs has been performed using high-performance liquid chromatography (HPLC) and mass spectrometry (MS) in a total or site-specific manner^24–29^. However, to date, the structural information on the FcγR glycosylation has been obtained using recombinant proteins produced by mammalian cell lines. Furthermore, the glycosylation patterns of the recombinant FcγRs depend on the expression vehicles^14,25^. To understand the molecular mechanisms of the FcγR-mediated functions better, from both immunological and therapeutic perspectives, it is crucial to elucidate the glycosylation profiles of the endogenous FcγRs.

To address this issue, as a first step, we performed site-specific *N*-glycosylation profiling of sFcγRIII purified from human serum. Plasma level of sFcγRIII is approximately 1 μg/mL in healthy individuals^30^. The vast majority of sFcγRIII molecules present in plasma are derived from neutrophils^31^, indicating that sFcγRIIIb is a dominant isoform in the serum. Using liquid chromatography (LC)-electrospray tandem mass spectrometry (MS/MS) analysis, we analyzed the site-specific *N*-glycosylation profile of the endogenous sFcγRIII, consisting of the extracellular domains released from the immune cell membranes by proteolytic cleavage.

## Results and Discussion

As previously reported^9^, sFcγRIIIb was purified from a pool of human serum by a series of chromatographic procedures. Consistent with the previous report^9^, we obtained a smear, instead of a band, of the sFcγRIIIb, which was stained with Coomassie Brilliant Blue (CBB), indicating that the serum sFcγRIIIb is highly glycosylated with considerable heterogeneity (Supplementary Fig. 1). According to the sandwich ELISA results, we obtained 100 μg of sFcγRIIIb from 2 L of human serum. Due to the limited sample availability, we performed only one round of sFcγRIIIb purification and site-specific glycosylation profiling.

**Figure 2:**
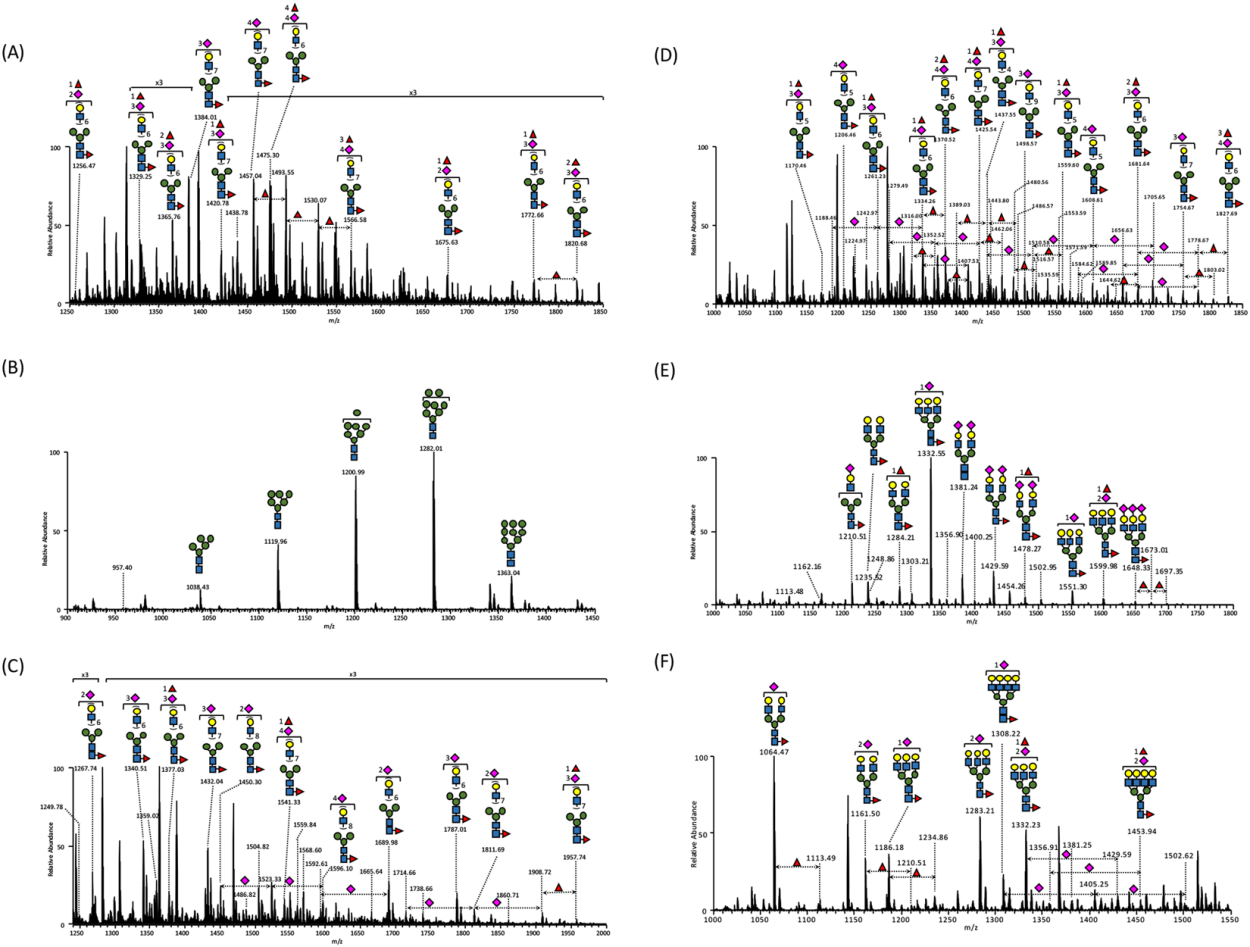
MS profiling of site-specific glycoforms of the serum sFcγRIIIb, covering six *N*-glycosylation sites. (**A**) Asn38, (**B**) Asn45, (**C**) Asn64, (**D**) Asn74, (**E**) Asn162, and (**F**) Asn169. The spectra are based on the averaged mass scans of the glycopeptides containing individual *N*-glycosylation sites, which eluted in time ranges of (**A**) 27.8–29.1 min, (**B**) 32.7–33.4 min, (**C**) 31.0–33.8 min, (**D**) 21.8–24.8 min, (**E**) 32.0–39.0 min, and (**F**) 44.0–50.0 min. The identities of Man, Gal, Fuc, and GlcNAc were inferred from the literature and are represented by symbols according to the Symbol Nomenclature for Glycans (SNFG) system^40^, the details of which can be found at NCBI (http://www.ncbi.nlm.nih.gov/books/NBK310273/): GlcNAc, ; Man, ; Gal, ; Neu5Ac, ; Fuc, .

We analyzed the purified serum sFcγRIIIb using LC-MS/MS after the GluC and chymotrypsin digestions, and identified and semiquantified (i) 14 glycoforms on Asn38, (ii) 6 glycoforms on Asn45, (iii) 30 glycoforms on Asn64, (iv) 45 glycoforms on Asn74, (v) 55 glycoforms on Asn162, and (vi) 15 glycoforms on Asn169 (Fig. 2, Table 1, and Supplementary Tables 1–6). Our data revealed that each *N*-glycosylation site of the serum sFcγRIIIb was modified in a distinct fashion in terms of number, composition, and variability of *N*-glycans.

**Table 1:** MS data obtained for glycopeptides in the chymotrypsin or GluC digests of human serum sFcγRIIIb.

Peptide Sequence	Enzyme	Site	M	[M + GlcNAc]^+^	[M + GlcNAc]^2+^
(Y)SPED**N**STQW(F)	Chymotrypsin	Asn38	1062.42	1266.51	—
(W)FH**N**ESLI(S)	Chymotrypsin	Asn45	858.42	1062.51	—
(F)IDAATV**N**DSGEY(R)	Chymotrypsin	Asn64	1253.54	1457.63	—
(Y)RCQT**N**L(S)	Chymotrypsin	Asn74	790.37	994.46	—
(D)SGSYFCRGLVGSK**N**VSSE(T)	GluC	Asn162	1932.9	2136.99	1069
(E)TV**N**ITITQGLA(V)	GluC	Asn169	1129.63	1333.72	—

^a^C, Carboxymethyl cysteine

**Figure 3:**
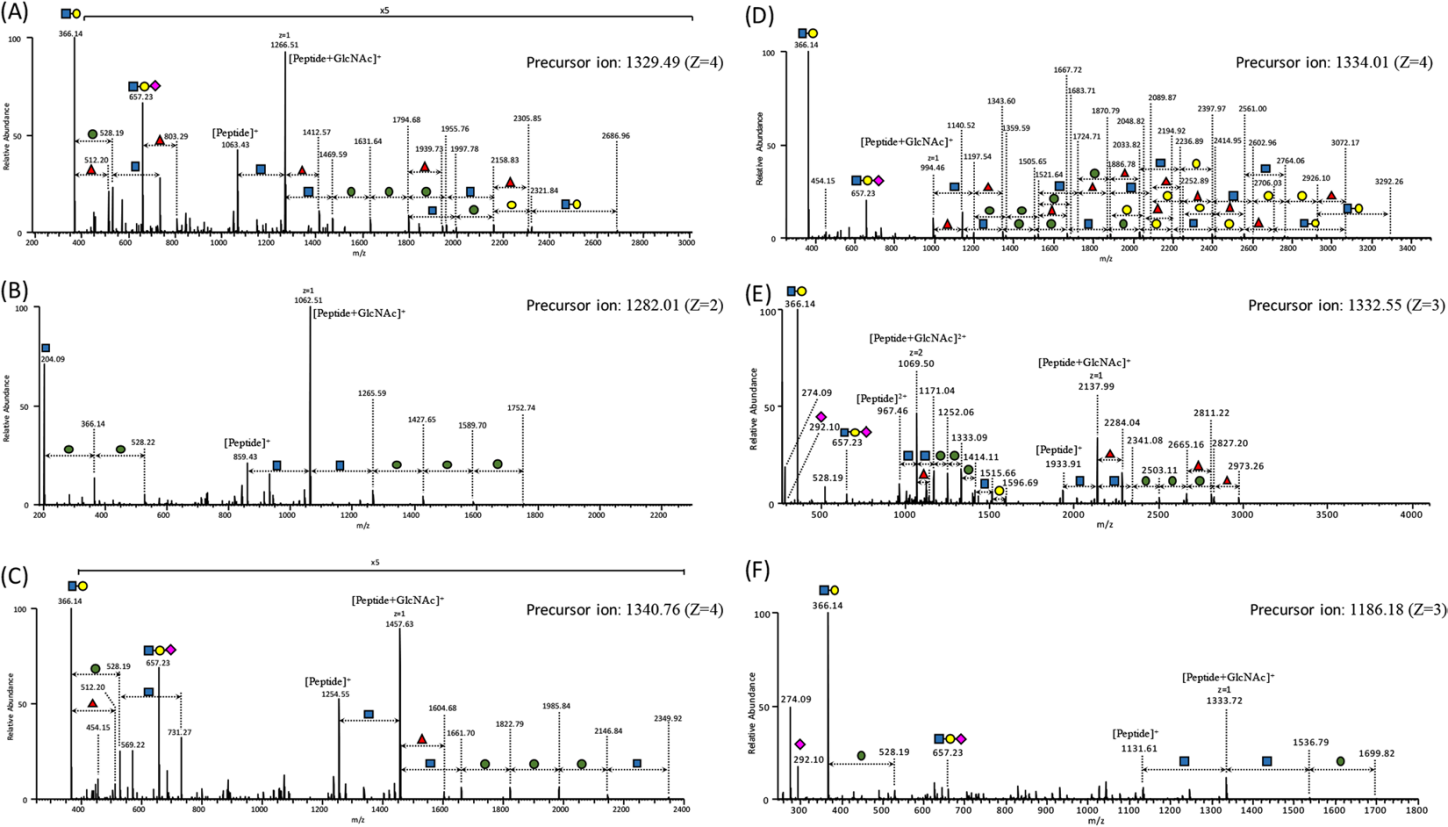
MS/MS spectra of the major glycoforms of glycopeptides covering six *N*-glycosylation sites. (**A**) Asn38, (**B**) Asn45, (**C**) Asn64, (**D**) Asn74, (**E**) Asn162, and (**F**) Asn169. The identities of Man, Gal, Fuc, and GlcNAc were inferred from the literature and are represented by symbols according to the Symbol Nomenclature for Glycans (SNFG) system^40^, the details of which can be found at NCBI (http://www.ncbi.nlm.nih.gov/books/NBK310273/): GlcNAc, ; Man, ; Gal, ; Neu5Ac, ; Fuc, .

In Fig. 3, the examples of MS/MS spectra obtained for the major glycoforms found on protease-digested glycopeptides with individual *N*-glycosylation sites are presented, revealing that all *N*-glycosylation sites, except Asn45, were modified with highly-branched sialyl glycans (Table 2). Furthermore, we identified more than one fucose residue in many *N*-glycans, suggesting the existence of the Lewis X or Lewis A structures in addition to core fucose modification. Our previous *N*-glycosylation profiling of the recombinant sFcγRIIIb expressed by baby hamster kidney (BHK) cells identified highly-branched sialyl *N*-glycans but not the non-reducing terminal fucose residues^27^. In contrast, the vehicle-specific *N*-glycan structures not found in the serum sFcγRIIIb are the following: Lewis X-containing *N*-glycans found in Chinese hamster ovary (CHO)-derived sFcγRIIIa^25^, LacdiNAc (GalNAcβ1-4GlcNAc)-containing glycans from the human embryonic kidney (HEK-293) cell-derived sFcγRIIIa^25^, poly-LacNAc (Galβ1-4GlcNAcβ1-4Galβ1-4GlcNAc)-containing glycans in human cell-derived sFcγRIIIa and sFcγRIIIb^24^, and galabiose-containing *N*-glycans in NS0-expressed sFcγRIIIa and sFcγRIIIb^28^. The present data thus indicate that the serum sFcγRIIIb exhibits different *N*-glycosylation profiles from those of the recombinant sFcγRIII glycoproteins.

**Table 2:** Major glycoforms of the individual *N*-glycosylation sites of human serum sFcγRIIIb.

Sites	Observed Mass (m/z)	z	Glycopeptide Mass	Glycan Mass	Glycoform^a^
Asn38	1328.74	4	5310.93	4248.5	dHex2 Hex9 HexNAc8 NeuNAc3
Asn45	1281.51	2	2561	1702.58	Hex8 HexNAc2
Asn64	1340.01	4	5356.01	4102.45	dHex1 Hex9 HexNAc8 NeuNAc3
Asn74	1330.54	4	5329.97	4539.61	dHex2 Hex9 HexNAc8 NeuNAc4
Asn162	1331.89	3	3554.5	2424.87	dHex1 Hex6 HexNAc5 NeuNAc1
Asn169	1185.84	3	3554.5	2424.87	dHex1 Hex6 HexNAc5 NeuNAc1

^a^Hex, hexose; HexNAc, *N*-acetylhexosamine; NeuNAc, *N*-acetylneuraminic acid; dHex, deoxyhexose.

Site-specific glycosylation information has been reported for recombinant sFcγRIIIb produced by BHK cells^26^, as well as for the recombinant sFcγRIIIa produced by HEK293T and CHO cells^25^. In Table 3, site-specific *N*-glycan classification of the human serum sFcγRIII is compared with those of the recombinant sFcγRIII glycoproteins. In the serum sFcγRIIIb, the Asn45 glycosylation site exclusively displays high-mannose-type glycans, whereas other glycosylation sites solely exhibit complex-type glycans. The glycans at Asn38, Asn74, Asn162, and Asn169 apt to be modified with complex-type oligosaccharides in the recombinant sFcγRIIIs as well. In the BHK-generated sFcγRIIIb, the major glycans associated with the Asn45 site were consistently shown to be of the high-mannose type, whereas Asn64 is inconsistently modified also by high-mannose-type oligosaccharides^26^. Considerable fractions of the Asn45 glycans were shown to be occupied by hybrid-type oligosaccharides in the recombinant sFcγRIIIa glycoproteins.

**Table 3:** Site-specific classification of *N*-glycans of the endogenous and recombinant sFcγRIIIb.

Sites	Serum sFcγRIIIb in this study	BHK-expressed sFcγRIIIb^26^	HEK293T-expressed sFcγRIIIa^25^	CHO-expressed sFcγRIIIa^25^
Asn38	Complex > 99%	Complex > 99%	—^*a*^	—
Asn 45	High-mannose > 99%	High-mannose 82%	Hybrid 16%	Hybrid 70%
		Complex 12%	Complex 84%	Complex 30%
Asn 64	Complex > 99%	High-mannose 41%	substitution	substitution
		Complex 47%		
Asn 74	Complex > 99%	Complex > 99%	Complex > 99%	Complex > 99%
Asn 162	Complex > 99%	Complex > 99%	Hybrid 25%	Complex > 99%
			Complex 75%	
Asn 169	Complex > 99%	Complex > 99%	Complex > 99%	—

^a^The glycopeptides were not observed under the LC-MS/MS conditions used in this study.

In general, the progression of *N*-glycan processing depends on the degree of exposure of the individual oligosaccharide moiety to the solvent, as demonstrated by the statistical analysis^32^: Highly accessible asparagine residues tend to be occupied by complex-type glycans, while less-exposed sites are frequently occupied by high-mannose-type and/or hybrid-type glycans. The solvent accessibility to the *N*-glycosylated asparagine residues in the crystal structure of sFcγRIIIb (PDB code: 1FNL)^33^ is as follows: Asn162 > Asn64 > Asn169 > Asn38 > Asn45 > Asn74. Therefore, the magnitude of *N*-glycan maturation cannot be simply ascribed to the solvent exposure of glycosylation sites in the native tertiary structure of a carrier protein estimated from the crystal structure.

Two alleles of human FcγRIIIb, NA1 and NA2, have been identified, and they differ in four amino-acid positions, which results in differences in the potential *N*-glycosylation site numbers: Asn45 and Asn64 glycosylation sites are not found in NA1 (Fig. 1)^34^. In agreement with the data presented here, FcγRIIIb on neutrophils from the NA2 donors was shown to be more reactive with concanavalin A than that obtained from the NA1 donors^35^. Homozygous NA2 individuals have a lower capacity to mediate phagocytosis than NA1 individuals, suggesting that FcγRIIIb-mediated immunological functions are negatively affected by the *N*-glycans at these non-conserved glycosylation sites^36^. Complement receptor 3 (CR3) was proposed to exhibit lectin-like activity and thereby interacts with the soluble and membrane-associated FcγRIIIb glycoproteins through their high-mannose-type oligosaccharides^26,37^. Based on our data, we propose that the interactions between FcγRIIIb and CR3 are exclusively mediated by the Asn45-associated glycans and specific for the NA2 allele.

Recently, Hayes *et al*.^15^ have reported a relationship between glycosylation profiles of human sFcγRIIIa produced by different vehicles and their IgG1-binding affinities, indicating that sialylated and/or multi-antennary *N*-glycans of sFcγRIIIa negatively contribute to the interactions with IgG1. Previously obtained structural and biochemical data suggest that the Asn45 and Asn162 glycans of FcγRIII are involved in the IgG1 interactions^12,17^. The present and previous site-specific glycosylation profiling studies demonstrate that the Asn45 site tends to display high-mannose-type *N*-glycans, whereas Ans162 is occupied by the complex-type glycans. Therefore, it is possible that the sialylation and multi-branching of the Asn162 glycans impair the IgG-FcγRIII interactions due to the negative steric effects. Reciprocally, our molecular dynamics simulation and crystallographic data obtained for the complexes formed between IgG1-Fc and sFcγRIIIa indicate that the core fucosylation of the IgG1-Fc glycan repels the Asn162 glycan of sFcγRIII, resulting in an increased conformational fluctuation of this *N*-glycan^38^. These data underscore the therapeutic significance of FcγRIII glycosylation, best illustrated by its Asn162 glycans, which play an important role in the promotion of ADCC by enhancing the interactions with the non-fucosyl IgG1^12,16^.

In previous studies, Asn162 of the recombinant sFcγRIIIa glycoproteins expressed by HEK293T and CHO cells were shown to be exclusively modified by biantennary complex-type glycans with partial sialylation^25^. In contrast, our results reveal that in human serum sFcγRIIIb, the major glycoforms at Asn162 are partially sialylated tri-antennary glycans, suggesting different functional glycosylation between native and recombinant FcγRIII glycoproteins. Moreover, an earlier study indicated that the endogenous FcγRIII exhibits cell-type-specific glycoforms based on the distinct lectin-binding properties^35^. Therefore, to optimize the design of therapeutic antibodies targeting FcγRIII displayed on specific cells, such possible variations of the receptor glycoforms should be considered. The data obtained in this study may assist the development of therapeutic antibodies with maximum efficacy in terms of effector functions mediated by the glycoforms of endogenous FcγRIII.

## Methods

### Purification of sFcγRIIIb from human serum

The sFcγRIIIb glycoprotein was purified from 2 L of pooled off-the-clot human serum (Access Biologicals) as previously described with modifications^9^. The initial purification step included precipitation with 40–60% saturated ammonium sulfate. The precipitate was re-solubilized in phosphate-buffered saline and then applied to a Blue Sepharose 6 Fast Flow column (GE Healthcare) to remove albumin. The flow-through fraction was initially fractionated using a Protein A Sepharose 4 Fast Flow column (GE Healthcare) and then by an anti-FcγRIII antibody (3G8)-conjugated sepharose column. The elution fraction was fractionated using a Protein G Sepharose 4 Fast Flow column followed by Superdex 200 16/60 GL Chromatographic Separation Column (GE Healthcare). Finally, the purified sFcγRIIIb glycoprotein was identified by sodium dodecyl sulfate-polyacrylamide gel electrophoresis followed by CBB staining (Fig. 2) and quantitated by sandwich ELISA as previously described^9^.

### Digestion and glycopeptide enrichment of serum sFcγRIIIb

The purified sFcγRIIIb glycoproteins (10 µg) were reduced in dithiothreitol (DTT; 25 mM) for 45 min at 60 °C and *S*-carbamidomethylated with iodoacetamide (42 mM) at room temperature for 30 min in the dark followed by quenching with DTT (10 mM). The denatured and *S*-carbamidomethylated sFcRγIIIb glycoprotein was incubated with endoproteinase GluC (0.5 μg; Thermo Fisher Scientific) in ammonium bicarbonate buffer (100 mM; pH 8.0) or chymotrypsin (Thermo Fisher Scientific) in Tris-HCl buffer (100 mM) containing calcium chloride (2 mM) at 37 °C for 20 h. Acetone-based glycopeptide enrichment method was used as described in a previous study^39^. The glycopeptides were precipitated with five-fold volume of ice-cold acetone followed by centrifugation at 12,000 × *g* for 10 min, and dissolved in formic acid (0.1%) for LC/MS.

### Site-specific glycosylation analysis by MS

Glycopeptides were analyzed by LC-MS/MS. HPLC was performed on an EASY-nLC 1000 (Thermo Fisher Scientific) equipped with an Acclaim PepMap 100 trapping column (75 × 20 mm, nanoViper; Thermo Scientific) and a Nano HPLC Capillary Column (75 × 120 mm, 3 μm, C18; Nikkyo Technos) at a flow rate of 0.3 µL/min. The eluents comprised 0.1% formic acid (A buffer) and 0.1% formic acid in acetonitrile (B buffer). The samples were eluted with a linear gradient from 0% to 35% B buffer over 60 min. MS analyses were performed using a Q Exactive mass spectrometer (Thermo Scientific) equipped with Nanospray Flex Ion Source (Thermo Scientific). The electrospray voltage was 2.0 kV, and the resolution was 70,000. Mass spectrometer was operated in positive ion mode and full mass spectra were acquired using an *m/z* range of 350–2000. Following every regular mass acquisition, we performed MS/MS acquisitions against the 10 most-intense ions using a data-dependent acquisition method with normalized collision energy of 27%. Product ion spectra of glycopeptides were manually selected based on the identification of oligosaccharide oxonium ions, with a characteristic *m/z* such as 204.09 (HexNAc) and 366.14 (HexNAc-Hex). The peptide and glycan masses of glycopeptides were deduced from the molecular masses of the peptide ion carrying a single *N*-acetylglucosamine, commonly considered more intense. Monosaccharide glycoform compositions were deduced using GlycoMod tool software. The remaining glycoforms were identified using the mass intervals between the glycoforms. The percentage distribution of the glycopeptides was calculated using the peak area of extracted ion chromatogram (XIC) and summed across all charge state of glycoforms. The most abundant ions were used for quantitative analysis.

## Electronic supplementary material

Supplementary information
