# Supplementary material for: Site-specific N-glycosylation analysis of soluble Fcγ receptor IIIb in human serum
Source: Sci Rep. 2018 Feb 9;8:2719. doi: 10.1038/s41598-018-21145-y (PMC5807427; doi:10.1038/s41598-018-21145-y)
Supplement: Supplementary file 1 — Supplementary information [file 41598_2018_21145_MOESM1_ESM.pdf]

Supplementary information

Site-specific *N*-glycosylation analysis of soluble Fcγ receptor IIIb in human serum

Hirokazu Yagi[1,a], Daisuke Takakura[2,a,b], Lubka T. Roumenina[3], Wolf Herman Fridman[3], Catherine Sautès-Fridman[3], Nana Kawasaki[2,*], and Koichi Kato[1,4,*]

[1] Faculty and Graduate School of Pharmaceutical Sciences, Nagoya City University, 3-1 Tanabe-dori, Mizuho-ku, Nagoya 467-8603, Japan
[2] Department of Medical Life Science, Graduate School of Medical Life Science, Yokohama City University, Suehiro-cho 1-7-29, Tsurumi-ku, Yokohama 230-0045, Japan
[3] UMRS1138, Université Paris Descartes, Université Pierre et Marie Curie, 15, rue de l'Ecole-de-Médecine 75270 Paris, France
[4] Institute for Molecular Science and Okazaki Institute for Integrative Bioscience, National Institutes of Natural Sciences, 5-1 Higashiyama Myodaiji, Okazaki 444-8787, Japan

[1]These authors contributed equally to this work.

[2] Current Address: Center for Integrated Medical Research, Keio University School of Medicine, 35 Shinanomachi, Shinjuku-ku, Tokyo 160-8582, Japan

[*]Corresponding author: Koichi Kato, Ph.D., Graduate School of Pharmaceutical Sciences, Nagoya City University, 3-1 Tanabe-dori, Mizuho-ku, Nagoya 467-8603, Japan and Okazaki Institute for Integrative Bioscience and Institute for Molecular Science, National Institutes of Natural Sciences, 5-1 Higashiyama Myodaiji, Okazaki 444-8787, Japan, TEL & FAX: +81-564-59-5225, e-mail: kkato@phar.nagoya-cu.ac.jp; and Nana Kawasaki, Ph.D., Department of Medical Life Science, Graduate School of Medical Life Science, Yokohama City University, Suehiro-cho 1-7-29, Tsurumi-ku, Yokohama 230-0045, Japan, TEL and FAX: +81-45-508-7667 e-mail: nana@yokohama-cu.ac.jp

Supplementary Figure

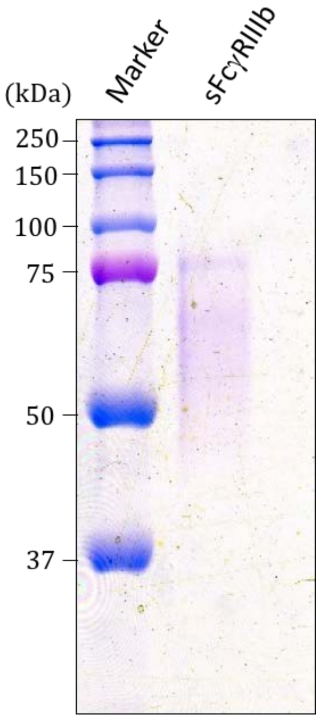

Supplemental Figure 1: SDS-PAGE of the sFcγRIIIb glycoprotein purified from human serum.

Supplemental Table 1: MS data of glycopeptides containing Asn38.

Composition[a]										
Hex	dHex	HexNAc	NeuNAc	Observed m/z	Z	Calculated mass	Theoretical mass	RT (min)	Peak area	Area (%)
9	1	8	2	1625.60	3	4873.78	4873.78	28.9	166704016	5.34
9	1	8	2	1219.45	4	4873.77	4873.78	29.1		
9	2	8	2	1674.29	3	5019.85	5019.84	28.8	149992822	4.8
9	2	8	2	1255.97	4	5019.85	5019.84	28.8		
9	1	8	3	1722.64	3	5164.90	5164.88	29.9	202382333	6.48
9	1	8	3	1292.23	4	5164.89	5164.88	29.8		
9	2	8	3	1771.32	3	5310.94	5310.94	29.1	415074618	13.29
9	2	8	3	1328.74	4	5310.93	5310.94	29.0		
9	3	8	3	1820.00	3	5456.98	5457.00	28.7	264755959	8.48
9	3	8	3	1365.26	4	5457.01	5457.00	28.8		
9	3	8	4	1438.03	4	5748.09	5748.09	29.1	194075442	6.22
9	4	8	4	1474.54	4	5894.13	5894.15	28.6	324272398	9.05
10	1	9	3	1383.51	4	5530.01	5530.01	28.5	290767409	9.31
10	2	9	3	1420.03	4	5676.09	5676.07	28.4	313027830	10.03
10	3	9	3	1456.54	4	5822.13	5822.13	28.1	129364104	4.14
10	1	9	4	1456.28	4	5821.09	5821.11	28.9	116964982	3.75
10	2	9	4	1492.80	4	5967.17	5967.17	28.8	235904254	9.56
10	3	9	4	1529.31	4	6113.21	6113.22	28.4	216653561	6.94
10	4	9	4	1565.83	4	6259.29	6259.28	27.8	121128470	2.62

[a] Hex, hexose; HexNAc, N-acetylhexosamine; NeuNAc, N-acetylneuraminic acid; dHex, deoxyhexose.

Supplemental Table 2: MS data of glycopeptides containing Asn45.

Composition[a]										
Hex	dHex	HexNAc	NeuNAc	Observed m/z	Z	Calculated mass	Theoretical mass	RT (min)	Peak area	Area (%)
4	0	2	0	957.40	2	1912.78	1912.79	33.2	48893172	0.27
5	0	2	0	1038.43	2	2074.84	2074.85	33.4	1397969634	7.63
6	0	2	0	1119.46	2	2236.90	2236.90	33.2	2859955726	15.61
6	0	2	0	746.64	3	2236.90	2236.90	33.4		
7	0	2	0	1200.49	2	2398.96	2398.95	33.0	5338038327	29.13
7	0	2	0	800.66	3	2398.96	2398.95	33.3		
8	0	2	0	1281.51	2	2561.00	2561.00	32.8	6453594224	35.22
8	0	2	0	854.68	3	2561.02	2561.00	32.8		
9	0	2	0	1362.54	2	2723.06	2723.06	32.7	2224417866	12.14
9	0	2	0	908.69	3	2723.05	2723.06	32.7		

[a] Hex, hexose; HexNAc, N-acetylhexosamine; NeuNAc, N-acetylneuraminic acid; dHex, deoxyhexose.

Supplemental Table 3: MS data of glycopeptides containing Asn64.

Composition[a]										
Hex	dHex	HexNAc	NeuNAc	Observed m/z	Z	Calculated mass	Theoretical mass	RT (min)	Peak area	Area (%)
7	1	6	1	1348.85	3	4043.53	4043.54	32.8	189975528	1.34
7	2	6	1	1397.54	3	4189.60	4189.60	32.6	18359711	0.13
7	1	6	2	1445.89	3	4334.65	4334.64	33.8	496186813	3.49
7	2	6	2	1494.57	3	4480.69	4480.69	33.6	92625723	0.65
8	1	7	1	1470.57	3	4408.69	4408.67	32.1	188954627	1.33
8	1	7	2	1567.60	3	4699.78	4699.77	33	442757469	3.12
8	2	7	2	1616.29	3	4845.85	4845.83	32.8	111215529	0.78
8	1	7	3	1664.63	3	4990.87	4990.86	33.9	998638733	7.03
8	1	7	3	1248.72	4	4990.85	4990.86	33.9		
8	2	7	3	1713.32	3	5136.94	5136.92	33.6	347672703	2.45
8	2	7	3	1285.24	4	5136.93	5136.92	33.6		
9	1	8	1	1592.28	3	4773.82	4773.80	31.5	287989605	2.03
9	1	8	1	1194.46	4	4773.81	4773.80	31.5		
9	1	8	2	1689.31	3	5064.91	5064.90	32.3	1624035961	11.43
9	1	8	2	1267.23	4	5064.89	5064.90	32.3		
9	2	8	2	1737.99	3	5210.95	5210.96	32	384758964	2.71
9	2	8	2	1303.75	4	5210.97	5210.96	32		
9	1	8	3	1786.34	3	5356.00	5355.99	33.3	1724480312	12.13
9	1	8	3	1340.01	4	5356.01	5355.99	33.2		
9	2	8	3	1835.03	3	5502.07	5502.05	32.8	704211875	4.95
9	2	8	3	1376.52	4	5502.05	5502.05	32.9		
10	1	9	1	1713.99	3	5138.95	5138.94	31.1	394748186	2.78
10	1	9	1	1285.74	4	5138.93	5138.94	31.1		
10	2	9	1	1762.68	3	5285.02	5284.99	31	86111204	0.61
10	2	9	1	1322.26	4	5285.01	5284.99	30.9		

10	1	9	2	1811.02	3	5430.04	5430.03	31.7	924656995	6.51
10	1	9	2	1358.51	4	5430.01	5430.03	31.9		
10	2	9	2	1859.71	3	5576.11	5576.09	31.5	259412461	1.83
10	2	9	2	1395.03	4	5576.09	5576.09	31.6		
10	1	9	3	1908.05	3	5721.13	5721.13	32.7	1173839573	8.26
10	1	9	3	1431.29	4	5721.13	5721.13	32.7		
10	2	9	3	1956.74	3	5867.20	5867.19	32.5	501765521	3.53
10	2	9	3	1467.81	4	5867.21	5867.19	32.4		
10	1	9	4	1504.06	4	6012.21	6012.22	33.6	452832641	3.19
10	2	9	4	1540.58	4	6158.29	6158.28	33.5	350023826	2.46
11	1	10	1	1377.02	4	5504.05	5504.07	30.7	188825220	1.33
11	2	10	1	1413.54	4	5650.13	5650.13	30.5	62601810	0.44
11	1	10	2	1449.80	4	5795.17	5795.16	31.3	617613416	4.35
11	2	10	2	1486.31	4	5941.21	5941.22	31.2	225248931	1.58
11	1	10	3	1522.57	4	6086.25	6086.26	31.9	610836902	4.30
11	2	10	3	1559.09	4	6232.33	6232.32	31.8	298731967	2.10
11	1	10	4	1595.35	4	6377.37	6377.35	33	305530518	2.15
11	2	10	4	1631.86	4	6523.41	6523.41	32.8	148105940	1.04

[a] Hex, hexose; HexNAc, N-acetylhexosamine; NeuNAc, N-acetylneuraminic acid; dHex, deoxyhexose.

Supplemental Table 4: MS data of glycopeptides containing Asn74.

Composition[a]										
Hex	dHex	HexNAc	NeuNAc	Observed m/z	Z	Calculated mass	Theoretical mass	RT (min)	Peak area	Area (%)
7	1	6	3	1388.53	3	4162.57	4162.56	24.6	97904434	0.84
7	2	6	3	1437.22	3	4308.64	4308.62	24.3	214056133	1.83
7	2	6	3	1078.16	4	4308.61	4308.62	24.5		
7	3	6	3	1485.90	3	4454.68	4454.68	24.2	147810762	1.27
8	1	7	2	1413.21	3	4236.61	4236.60	23.0	93128133	0.80
8	1	7	2	1060.16	4	4236.61	4236.60	23.0		
8	2	7	2	1461.89	3	4382.65	4382.66	22.9	33439711	0.29
8	1	7	3	1510.24	3	4527.70	4527.70	23.7	313338251	2.69
8	1	7	3	1132.93	4	4527.69	4527.70	24.1		
8	2	7	3	1558.93	3	4673.77	4673.76	23.7	412487583	3.54
8	2	7	3	1169.44	4	4673.73	4673.76	23.5		
8	1	7	4	1607.27	3	4818.79	4818.79	24.7	511791659	4.39
8	1	7	4	1205.71	4	4818.81	4818.79	24.8		
8	2	7	4	1655.96	3	4963.85	4964.85	24.7	882519376	7.56
8	2	7	4	1242.22	4	4964.85	4964.85	24.5		
8	3	7	4	1704.65	3	5110.93	5110.91	24.3	801035217	6.87
8	3	7	4	1278.74	4	5110.93	5110.91	24.3		
8	4	7	4	1753.33	3	5256.97	5256.97	24.2	303850296	2.60
8	4	7	4	1315.25	4	5256.97	5256.97	24.3		
9	1	8	2	1534.92	3	4601.74	4601.73	22.6	392439262	3.36
9	1	8	2	1151.44	4	4601.73	4601.73	22.6		
9	2	8	2	1583.61	3	4747.81	4747.79	22.4	183232719	1.57
9	2	8	2	1187.96	4	4747.81	4747.79	22.4		
9	1	8	3	1631.95	3	4892.83	4892.83	22.5	485696549	4.16
9	1	8	3	1224.22	4	4892.85	4892.83	22.5		
9	2	8	3	1680.64	3	5038.90	5038.89	23.3	602971371	5.17
9	2	8	3	1260.73	4	5038.89	5038.89	23.2		
9	2	8	4	1777.67	3	5329.99	5329.98	23.9	685768564	5.88
9	2	8	4	1333.50	4	5329.97	5329.98	23.9		
9	3	8	4	1826.35	3	5475.02	5476.04	23.7	495271192	4.25
9	3	8	4	1370.02	4	5476.05	5476.04	23.7		
9	4	8	4	1406.53	4	5622.09	5622.10	23.4	204364646	1.75
10	1	9	2	1656.63	3	4966.87	4966.87	22.3	230290297	1.97
10	1	9	2	1242.72	4	4966.85	4966.87	22.3		
10	2	9	2	1705.32	3	5112.94	5112.92	22.4	171187733	1.47
10	2	9	2	1279.24	4	5112.93	5112.92	22.3		
10	1	9	3	1753.66	3	5257.96	5257.96	23.1	363229744	3.11
10	1	9	3	1315.50	4	5257.97	5257.96	23.1		
10	2	9	3	1802.35	3	5404.03	5404.02	23.0	497285344	4.26
10	2	9	3	1352.01	4	5404.01	5404.02	22.9		
10	3	9	3	1388.53	4	5550.09	5550.08	22.7	219924500	1.89
10	4	9	3	1425.04	4	5696.13	5696.14	22.4	91086177	0.78
10	1	9	4	1388.27	4	5549.05	5549.06	23.9	234478727	2.01
10	2	9	4	1424.79	4	5695.13	5695.11	23.7	328016590	2.81
10	3	9	4	1461.30	4	5841.17	5841.17	23.4	306940819	2.63
10	4	9	4	1497.82	4	5987.25	5987.23	23.3	186786196	1.60
11	1	10	2	1334.01	4	5332.01	5332.00	22.2	236952167	2.03

11	2	10	2	1370.52	4	5478.05	5478.06	21.9	24131769	0.21
11	1	10	3	1406.78	4	5623.09	5623.09	22.9	203029001	1.74
11	2	10	3	1443.30	4	5769.17	5769.15	22.6	320890252	2.75
11	3	10	3	1479.81	4	5915.21	5915.21	22.6	144674005	1.24
11	1	10	4	1479.56	4	5914.21	5914.19	23.6	169558216	1.45
11	2	10	4	1516.07	4	6060.25	6060.25	23.4	208482170	1.79
11	3	10	4	1552.58	4	6206.29	6206.30	23.1	145437052	1.25
11	4	10	4	1589.10	4	6352.37	6352.36	23.0	81241127	0.70
12	2	11	2	1461.80	4	5843.17	5843.19	22.0	32172360	0.28
12	1	11	2	1425.54	4	5698.13	5697.13	21.8	33736521	0.29
12	1	11	3	1498.07	4	5988.25	5988.23	22.6	129559872	1.11
12	2	11	3	1534.58	4	6134.29	6134.28	22.5	115862175	0.99
12	3	11	3	1571.09	4	6280.33	6280.34	22.3	79718739	0.68
12	1	11	4	1570.84	4	6279.33	6279.32	23.2	100434912	0.86
12	2	11	4	1607.35	4	6425.37	6425.38	23.1	84361468	0.72
12	3	11	4	1643.87	4	6571.45	6571.44	23.0	66443464	0.57

[a] Hex, hexose; HexNAc, N-acetylhexosamine; NeuNAc, N-acetylneuraminic acid; dHex, deoxyhexose.

Supplemental Table 5: MS data of glycopeptides containing Asn162

Composition[a]										
Hex	dHex	HexNAc	NeuNAc	Observed m/z	Z	Calculated mass	Theoretical mass	RT (min)	Peak area	Area (%)
4	0	3	0	1064.46	3	3190.36	3190.35	32.9	81754431	0.07
4	1	3	0	1113.14	3	3336.40	3336.41	32.7	2439094273	1.98
4	2	3	0	1161.83	3	3482.47	3482.46	32.6	1364814815	1.11
4	0	3	1	1161.49	3	3481.45	3481.44	35.1	297305933	0.24
4	1	3	1	1210.18	3	3627.52	3627.50	34.9	6700577049	5.43
4	2	3	1	1258.86	3	3773.56	3773.56	34.8	1005756836	0.81
4	1	4	0	1180.84	3	3539.50	3539.49	32.6	715582489	0.58
4	2	4	0	1229.53	3	3685.57	3685.54	32.4	58487149	0.05
4	1	4	1	1277.87	3	3830.59	3830.58	34.8	372797878	0.30
4	2	4	1	1326.56	3	3976.66	3976.64	32.3	121807573	0.10
5	0	4	0	1186.16	3	3555.46	3555.48	32.6	93478633	0.08
5	1	4	0	1234.85	3	3701.53	3701.54	32.3	6992302771	5.66
5	2	4	0	1283.54	3	3847.60	3847.60	32.2	3100303295	2.51
5	3	4	0	1332.23	3	3993.67	3993.65	32.1	285573226	0.23
5	0	4	1	1283.20	3	3846.58	3846.58	34.7	344623199	0.28
5	1	4	1	1331.89	3	3992.65	3992.63	34.2	45866045269	37.15
5	2	4	1	1380.57	3	4138.69	4138.69	33.9	8831361754	7.15
5	2	4	1	1035.68	4	4138.69	4138.69	34.3		
5	3	4	1	1429.25	3	4284.73	4284.75	33.7	254673787	0.21
5	3	4	1	1072.19	4	4284.73	4284.75	33.8		
5	0	4	2	1380.23	3	4137.67	4137.67	36.8	120653575	0.10
5	1	4	2	1428.92	3	4283.74	4283.73	36.8	8258315122	6.70
5	2	4	2	1477.60	3	4429.78	4429.79	36.7	1975294575	1.60
5	3	4	2	1526.29	3	4575.85	4575.85	36.6	110808168	0.09
3	1	5	0	1194.51	3	3580.51	3580.51	32.8	190894570	0.15
4	1	5	0	1248.53	3	3742.57	3742.57	32.6	1524481114	1.24
5	1	5	0	1302.55	3	3904.63	3904.62	32.5	2517287322	2.04
5	2	5	0	1351.23	3	4050.67	4050.68	32.2	98463958	0.08
5	1	5	1	1399.58	3	4195.72	4195.71	34.4	1200208695	0.97
5	1	5	1	1049.94	4	4195.73	4195.71	34.5		
5	2	5	1	1448.26	3	4341.76	4341.77	33.8	39425663	0.03
5	1	5	2	1496.61	3	4486.81	4486.81	36.6	232729376	0.19
5	1	5	2	1122.71	4	4486.81	4486.81	36.6		
6	1	5	0	1356.57	3	4066.69	4066.67	32.1	1485836877	1.20
6	1	5	0	1017.68	4	4066.69	4066.67	32.1		
6	2	5	0	1405.25	3	4212.73	4212.73	32.1	514927912	0.42
6	2	5	0	1054.19	4	4212.73	4212.73	32.1		
6	3	5	0	1453.94	3	4358.80	4358.79	32.0	68042200	0.06
6	1	5	1	1453.60	3	4357.78	4357.77	33.7	4328762403	3.51
6	2	5	1	1502.28	3	4503.82	4503.82	33.7	1593214301	1.29
6	3	5	1	1550.97	3	4649.89	4649.88	33.6	249234383	0.20
6	1	5	2	1550.63	3	4648.87	4648.86	36.3	7039045342	5.71
6	1	5	2	1163.22	4	4648.85	4648.86			
6	2	5	2	1599.31	3	4794.91	4794.92	36.2	3134705437	2.54
6	2	5	2	1199.74	4	4794.93	4794.92	36.1		
6	3	5	2	1648.00	3	4940.98	4940.98	36.0	287636414	0.23
6	3	5	2	1236.25	4	4940.97	4940.98	36.1		
6	1	5	3	1647.66	3	4939.96	4939.96	38.6	2731454530	2.21
6	1	5	3	1236.00	4	4939.97	4939.96	38.6		

6	2	5	3	1696.35	3	5086.03	5086.02	38.4	1279788762	1.04
6	2	5	3	1272.51	4	5086.01	5086.02	38.4		
6	3	5	3	1745.03	3	5232.07	5232.07	39.0	130801324	0.11
6	3	5	3	1309.02	4	5232.05	5232.07	38.3		
7	1	6	0	1478.28	3	4431.82	4431.80	32.0	355652226	0.29
7	1	6	0	1108.96	4	4431.81	4431.80	32.0		
7	2	6	0	1526.96	3	4577.86	4577.86	31.9	100248901	0.08
7	2	6	0	1145.47	4	4577.85	4577.86	31.9		
7	3	6	0	1575.64	3	4723.90	4723.92	31.9	6350773	0.01
7	1	6	1	1575.31	3	4722.91	4722.90	33.5	1027888030	0.83
7	1	6	1	1181.73	4	4722.89	4722.90	33.5		
7	2	6	1	1623.99	3	4868.95	4868.96	33.5	541474311	0.44
7	2	6	1	1218.24	4	4868.93	4868.96	33.5		
7	3	6	1	1672.68	3	5015.02	5015.01	33.4	81411616	0.07
7	3	6	1	1254.76	4	5015.01	5015.01	33.4		
7	1	6	2	1672.34	3	5014.00	5013.99	35.9	1570816281	1.27
7	1	6	2	1254.51	4	5014.01	5013.99	35.9		
7	2	6	2	1721.03	3	5160.07	5160.05	35.7	807058547	0.65
7	2	6	2	1291.02	4	5160.05	5160.05	35.8		
7	3	6	2	1769.71	3	5306.11	5306.11	35.7	125987218	0.10
7	3	6	2	1327.54	4	5306.13	5306.11	35.7		
7	1	6	3	1769.37	3	5305.09	5305.09	37.7	451211544	0.37
7	1	6	3	1327.28	4	5305.09	5305.09	37.8		
7	2	6	3	1363.80	4	5451.17	5451.15	37.7	98067894	0.08
7	3	6	3	1400.31	4	5597.21	5597.21	37.9	15578958	0.01
8	1	7	1	1697.02	3	5088.04	5088.03	33.4	124357570	0.10
8	2	7	1	1745.70	3	5234.08	5234.09	33.3	103461484	0.08
8	2	7	1	1309.53	4	5234.09	5234.09	33.2		

[a] Hex, hexose; HexNAc, N-acetylhexosamine; NeuNAc, N-acetylneuraminic acid; dHex, deoxyhexose.

Supplemental Table 6: MS data of glycopeptides containing Asn169.

Composition[a]										
Hex	dHex	HexNAc	NeuNAc	Observed m/z	Z	Calculated mass	Theoretical mass	RT (min)	Peak area	Area (%)
5	1	4	1	1064.13	3	3189.37	3189.37	45.8	4296112999	27.50
5	1	4	2	1161.16	3	3480.46	3480.46	47.7	1479571470	9.47
5	2	4	1	1112.82	3	3335.44	3335.43	45.6	292621389	1.87
5	2	4	2	1209.85	3	3626.53	3626.52	47.4	141739144	0.91
6	1	5	1	1185.84	3	3554.50	3554.50	45.4	1668853370	10.68
6	1	5	2	1282.87	3	3845.59	3845.60	47.2	2753744451	17.63
6	1	5	3	1379.90	3	4136.68	4136.69	49.8	188173428	1.20
6	2	5	1	1234.53	3	3700.57	3700.56	45.2	417353757	2.67
6	2	5	2	1331.56	3	3991.66	3991.65	46.9	2249414981	14.40
6	2	5	3	1428.59	3	4282.75	4282.75	49.8	199291411	1.28
7	1	6	1	1307.55	3	3919.63	3919.63	44.9	784317769	5.02
7	1	6	2	1404.58	3	4210.72	4210.73	46.3	555865524	3.56
7	1	6	3	1501.62	3	4501.84	4501.82	48.5	62166162	0.40
7	2	6	1	1356.24	3	4065.70	4065.69	44.4	214473841	1.37
7	2	6	2	1453.27	3	4356.79	4356.79	46.2	318548059	2.04

[a] Hex, hexose; HexNAc, N-acetylhexosamine; NeuNAc, N-acetylneuraminic acid; dHex, deoxyhexose.
